# Comparison of Survival Outcomes between Early Breast Cancer Patients who Underwent Mastectomy and Patients Treated by Breast Conserving Therapy: A Meta Analysis

**DOI:** 10.24248/eahrj.v6i1.672

**Published:** 2022-07

**Authors:** Astère Manirakiza, Laurent Irakoze, Sébastien Manirakiza

**Affiliations:** a Ecole Doctorale de l'Université du Burundi; b University Teaching Hospital of Kamenge; c University of Burundi

## Abstract

**Background:**

Early stage of breast cancer requires mastectomy or breast conserving therapy. However, there are disagreements regarding the outcome of these two types of therapies in term of overall survivals.

**Objectives:**

The first aim of this meta-analysis was to assess the overall survival between patients who underwent mastectomy and those treated by breast conserving therapy. The second was to evaluate the influence of the follow up period on overall survival between the patients who benefited mastectomy and those who under went breast conservative therapy.

**Methods:**

We systematically searched on PubMed and Cochrane library all published randomized trials comparing mastectomy with breast conserving therapy and assessing overall survival.

**Results:**

Using dichotomous data, there was not a significant difference between mastectomy and BCT (OR:0.99; 95% CI:0.93-1.06; P:0.86). This was the same in subgroup analysis based on period of follow up. Their ORs and CI were (OR:0.97; 95% CI:0.81-1.18; P:0.79), (OR:1.01; 95% CI:0.90-1.13; P:0.87) and (OR:1.04; 95% CI:0.93-1.16; P:0.46) respectively for up to 5 years or less, between 5 and 10 years and more than 10 years of follow up. Using generic inverse variance, there was no significant difference between mastectomy and BCT, (HR:1.01; 95% CI:0.98-1.04; P:0.71). In subgroup analysis based on period of follow up, there was no significant difference between mastectomy and BCT. Their HRs, CI and P-value were (HR:1.01; 95% CI:0.951-1.07; P:0.79), (HR:0.98; 95% CI:0.92-1.04; P:0.51) and (HR:1.02; 95% CI:0.97-1.07; P:0.40) respectively for up to 5 years or less, between 5 and 10 years and more than 10 years of follow up.

**Conclusion:**

This meta-analysis demonstrated that there was no significant difference between patients with early stage breast cancer when they are treated by mastectomy or breast consevative therapy in term of overall survival. Additionnally, the follow up period had no any influence on the both types of surgery in term of overall survival. Therefore, we suggest that breast conservative therapy or mastectomy should be discussed between the care team and the patient, taking into account the financial means available to the patient, especially in low-income countries, the benefits of the surgery and the patient's choices.

## BACKGROUND

Breast cancer is one of the most common cancers worldwide. It is the leadingin female cancer in term of incidence and the second in term of mortality.^1^ Patients with early stage of breast cancer undergo either mastectomy or breast conserving therapy (BCT) followed by radiation therapy with preferences for the second choose.^2^ Several studies have compared the overall survival (OS) between patients treated by mastectomy with those underwent breast conserving therapy. Most of them found no significant difference between the two types of surgery regarding the overall survival but others found that the breast conserving therapy is the best and was some time advised to patients.^2-4^ This was also effective in one meta-analysis performed on patients with locally advanced breast cancer after good response to neoadjuvant chemotherapy where BCT was a safe surgery for patients and had good response.^5^

However, two recent meta-analyses, one using population-based studies and another randomized controlled trials concluded that mastectomy provides better OS than breast conserving surgery in women with early breast cancer.^6,7^ In these meta-analyses, both considered hazard ratio estimates for overall survival and 95% Confidence Interval (CI) as one of the inclusion criterions. Another meta-analysis performed with non-randomised studies reported that the 3 year or 5 year overall survival, was not statistically different between the BCT group and the mastectomy group.^8^ For this meta-analysis, the included studies reported the outcome as dichotomous data.

It is possible to analyse time-to-event data as dichotomous data (data from each intervention arm of each study are provided in a 2 × 2 table) even though the most appropriate way of summarising time-to-event data is to use methods of survival analysis and express the intervention effect as a hazard ratio as clarified by several studies.^9,10^

To address the divergences raised above, we conducted a meta-analysis of randomised trials using reported outcomes as dichotomous data or as hazard ratios. The objective of this meta-analysis was to comprehensively assess OS between patients with early-stage breast cancer who underwent mastectomy and those treated with breast-conserving therapy. Furthermore, it was to assess the influence of follow-up period and the effect of using dichotomous and generic inverse variances (data from each intervention group are provided as summary statistics) on OS.

## METHODS

### Study Selection and Data Extraction

To be included in this meta-analysis, studies should be published in English, randomized and comparing at least mastectomy with breast conserving therapy. Moreover, their outcomes should be reported in terms of overall survival (OS)and expressed either in Hazard Ratio (HR) or presented in dichotomous form.

The PubMed and Cochrane Library databases were searched for relevant papers up to 24^th^ October 2019. The search MeSH key words were ((Breast cancer) AND mastectomy) AND lumpectomy).

### Study Quality and Risk of Bias Assessment

There are many tools to assess the risk of reporting biases in studies even though they have several limitations.^11,12^ In this study, we adopted the revised Cochrane risk-of-bias tool for randomized trials (RoB 2), updated on 22^nd^ August 2019. It considers the risk of bias in the findings of any type of randomized trial and it assess the bias related to randomisation process, deviations from intended interventions, missing outcome data, measurement of the outcome and selection of the reported result.^13^

### Statistical Analysis

This study was assessed at two levels. The first was using dichotomous data and Odd Ratio (OR) with 95% confident interval (CI). The second was using life table data and Hazard Ratio (HR) with 95%CI. For the data reported as life table, they were adjusted and converted into HRs with their standard errors (SEs) by using the tool proposed by Tierney JF and his colleagues.^10^ In both cases, heterogeneity among studies was significant whether I^2^ > 50% with P<0.1 to 40%.^12^ Review Manager (RevMan) [Computer program]. Version 5.3. Copenhagen: The Nordic Cochrane Centre, The Cochrane Collaboration, 2014 was used for all statistical analyses. In both cases, we performed subgroups analysis to compare the OS in patients underwent mastectomy and those treated by BCT according to the period of follow up. The comparison was done between OS following the follow up period.

## RESULTS

**FIGURE 1:**
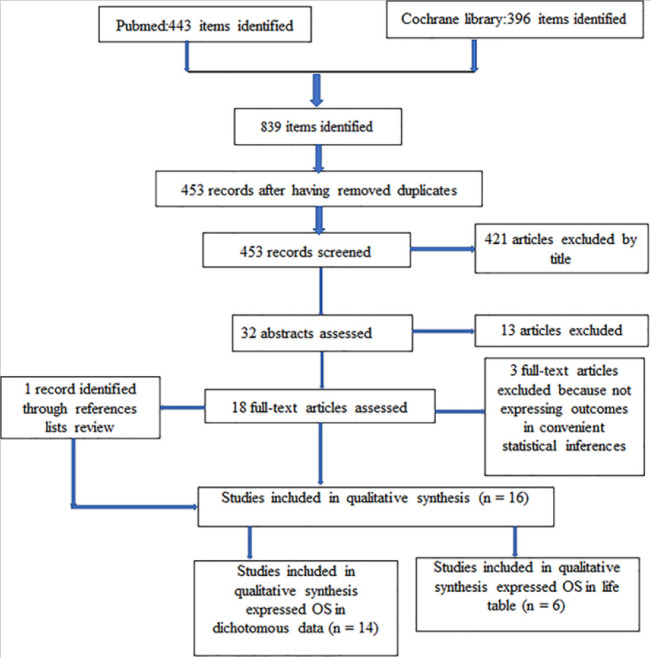
Pisma Flow Diagram

A total of 839 articles were identified in two online databases searched. After removing duplicates, we screened 453 articles. Only 32 abstracts were assessed after removing some papers by title. Eighteen papers were fully evaluated. During this process, three articles were removed but simultaneously another paper was identified through references list. Finally, 16 studies^14-29^ were included in the meta-analysis. Of them, 14 papers were suitable for dichotomous, 6 for generic inverses variances. Four studies were common for both types of data (figure 1). All studies compared at least mastectomy with breast conserving therapy. Stage I and II were found in all studies. The follow up period varied from 1 to 30 years. Studies characteristics were resumed in table 1.

**TABLE 1:** Studies Characteristics

Author & publication year	Interventions	Treatment after lumpectomy	Major inclusion criterion	Assessment period	Participants	
					MT	BCT
Veronesi U 1990	Classic Halsted mastectomy versus quadrantectomy, axillary dissection & radiotherapy on the ipsilateral breast	• Radiotherapy to the ipsilateral breast (50 Gy with high energy plus 10 Gy as a boost with orthovoltage) • Cyclophosphamide, methotrexate, fluorouracil)	Patients (< 70 years old), tumour <2 cm, no palpable axillary nodes, Stage I; T<2 cm; N0–1	10 & 13 yrs	349	352
Fisher B 1985	Total mastectomy, segmental mastectomy alone or segmental mastectomy followed by breast radiation	• A minimum of 5000 rad	Tumour size ≤ 4cm; no palpable axillary nodes, Stage I, II (Tl,2, N0,1, M0)	1,2,3,4 & 5 yrs	586	632
Litiere S 2012	Breast-conserving therapy versus modified radical mastectomy	• Whole breast radiotherapy & a tumour-bed boost (50 Gy in 5 weeks) with an additional boost dose of 25 Gy directed to the lumpectomy site	Tumours ≤ 5 cm, axillary node negative or positive disease carcinoma, Stage I or II disease	3,6,9,12,15,18,21,24,27 & 30 yrs	420	448
Jacobson JA 1995	Breast-conservation therapy versus mastectomy	• Radiation in an isodose of 4500 to 5040 cGy to the whole breast, fractioned in 180 cGy five days per week	Clinical stage I or II (T1 or T2, which included tumours ≤ 5 cm; NO or NI; M0) invasive carcinoma of the breast	3,6,9,12 & 15 yrs	116	121
Lee HD 1997	Modified radical mastectomy versus breast conserving therapy	• Radiotherapy (4 or 6 MeV) on the entire breast & supraclavicular fossa. Boost doses to the primary tumour site (9-15 MeV electron). • CMF (cyclophosphamide, methotrexate, and fluorouracil)	Stage I and II breast cancer with primary tumours ≤ 4 cm	6,12,18,24,30,36,42 & 48 months	111	76
Voogd AC 2001	Breast conservation versus modified radical mastectomy	• Whole breast irradiation (within 2-6 weeks of surgery), 50 Gy and an additional booster dose to the tumour bed.	Stage I and II breast, no age limit	1,2,3,4,5, 6 7,8,9 & 10 yrs	893	879
Sarrazin D 1989	Tumorectomy and breast irradiation versus modified radical mastectomy.	• 45 Gy in 18 fractions (4 fractions of 2.5 Gy/week) over one month. A booster dose of 15 Gy in 6 fractions over 10 days	Stage I or II (Tl–2 N0–1 M0) breast cancer, < 70 years old	2,4,6,8 & 10 yrs	91	88
Fisher B 1995	Total mastectomy versus lumpectomy	• Breast irradiation	Negative or positive axillary nodes & tumours ≤4 cm (stage I and II breast cancer)	2,4,6,8, 10 & 12	692	714
Simone NL 2012	Total mastectomy versus BCT	• 1,500-2,000 cGy boost to the tumour bed • Cyclophosphamide and doxorubicin	Invasive breast tumours ≤5 cm, clinically negative or positive axillary lymph nodes	5,10,15,20,25 & 30 yrs	116	121
van Dongen JA 1992	Modified radical mastectomy versus breast conserving therapy	• Radiotherapy to the breast (50 Gy in 5 weeks and a boost with iridium implant of 25 Gy)	TNM stage I and II	2,4,6,8, 10 & 12 yrs	424	455
Fisher B 1989	Total mastectomy versus lumpectomy	• Radiation (50 Gy)	Stage I, II; tumour ≤4 cm, Tl, 2, NO, NI, M0	1,2,3,4,5,6,7 & 8 yrs	590	629
Poggi MM 2003	Mastectomy versus Breast Conservation Therapy	• Radiation boost of 1500-2000 cGy to the tumour bed	Stage I or Stage II (T1 or T2; NO or NI; M0)	3,6,9,12,15,18,21,24 & 27 yrs	116	121
Lichter AS 1992	Mastectomy versus excisional biopsy (lumpectomy)	• A boost to the tumour bed using either an iridium 1 implant or an electron beam for an additional 1,500 to 2,000 cGy • Doxorubicin and cyclophosphamide	Stage TI or T2, NO or N1 invasive carcinoma of the breast	12,24,36,48,60,72,84,96,108 & 120 months	116	121
Blichert-Toft M 2008	Breast conserving surgery versus mastectomy	• Radiation (50 Gy in 25 fractions in 5 weeks) • nTumour bed received a boost dose of 10-25 Gy in 5-12 fractions •bCMF (Cyclophosphamide, Methotrexate)	Tumour ≤ 50 mm, One-sided, unifocal, <70 years old	5, 10, 15 & 20 yrs	350	381
van Dongen JA 2000	Breast-Conserving Therapy versus Mastectomy	• Radiotherapy to the breast • Booster dose of 25 Gy to (50 Gy over a 5-wee the lumpectomy site • Cyclophosphamide, methotrexate, and 5-fluorouracil	Tumours ≤5 cm	2,4,6,8,10,12,14,16 & 18 yrsk	420	448
Fisher B 2002	Total mastectomy versus lumpectomy	• 50 Gy of radiation	Tumours ≤ 4 cm, negative or positive axillary lymph nodes (stage I or II)	4,8,12,16 & 20 yrs	589	628

### Overall Survival.

### Outcome in Dichotomous Data

**FIGURE 2:**
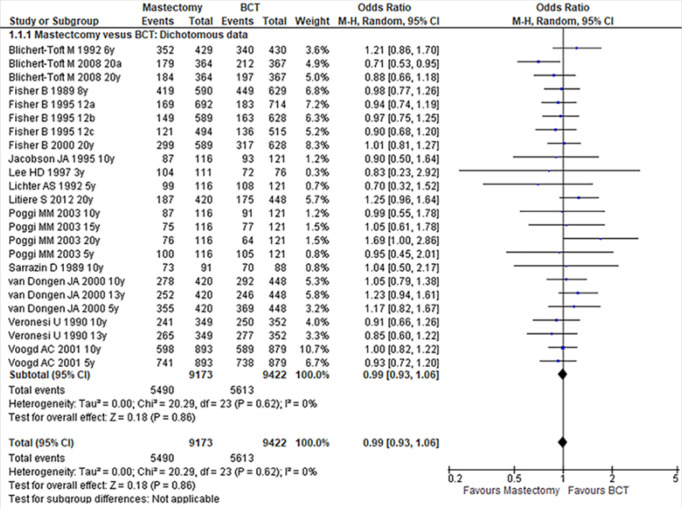
Forest Plot Comparing Mastectomy with BCT in Dichotomous Setting

The OS reported as rate was available in 14 studies. In this case, it is suggested that meta-analysis should be performed using dichotomous type. Therefore, in this study, we found no significant difference between mastectomy and BCT, (OR:0.99; 95% CI:0.93-1.06; P:0.86). There was no evidence of significant heterogeneity across studies included, (I^2^:0%, P:0.62), as shown in figure 2.

**FIGURE 3:**
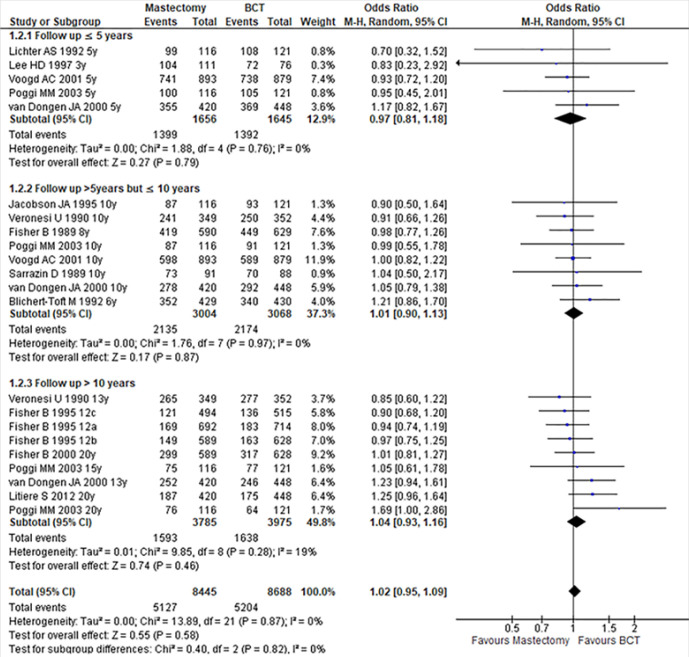
Forest Plot Comparing Mastectomy with BCT in Follow Up Period Subgrouping

In subgroups analysis, there was also no significant difference according to the follow-up period, whether for less than or equal to 5 years, between 5 and 10 years or more than 10 years. Their ORs and CIs were respectively (OR:0.97; 95% CI:0.81-1.18; P:0.79), (OR:1.01; 95% CI:0.90-1.13; P:0.87) and (OR:1.04; 95% CI:0.93-1.16; P:0.46). In the three cases, there was no evidence of significant heterogeneity across studies. Their I^2^ and P-value are (I^2^:0%, P:0.76); (I^2^:0%, P:0.97); (I^2^:19%, P:0.28) respectively for up to 5 years or less, between 5 and 10 years and more than 10 years (figure 3).

### Outcome in Generic Inverse Variance

**FIGURE 4:**
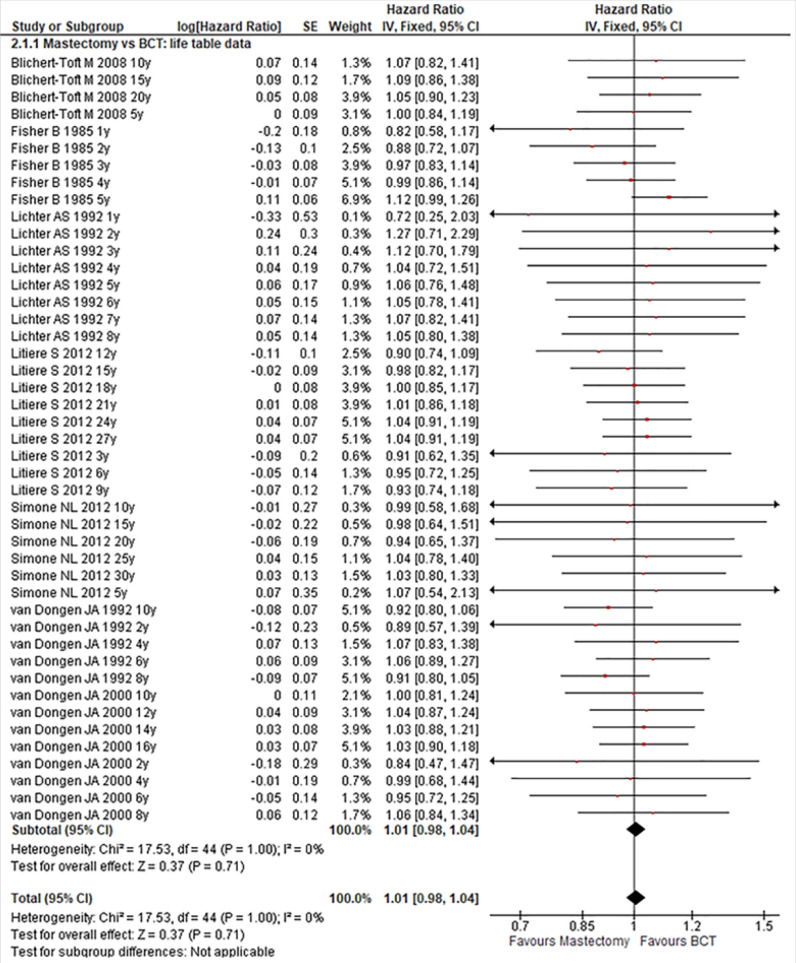
Forest Plot Comparing Mastectomy with BCT in Generic Inverses Variances Setting

The OSs reported as HRs were available in six studies. Performing meta-analysis by log (HR) with SEs, we did not find any evidence of significant difference between the patients treated by mastectomy compared with those treated by BCT in term of OS, (HR:1.01; 95% CI:0.98-1.04; P:0.71). Across studies, there was no evidence of heterogeneity, (I^2^: 0%, P:1.00) as shown in figure 4.

**FIGURE 5:**
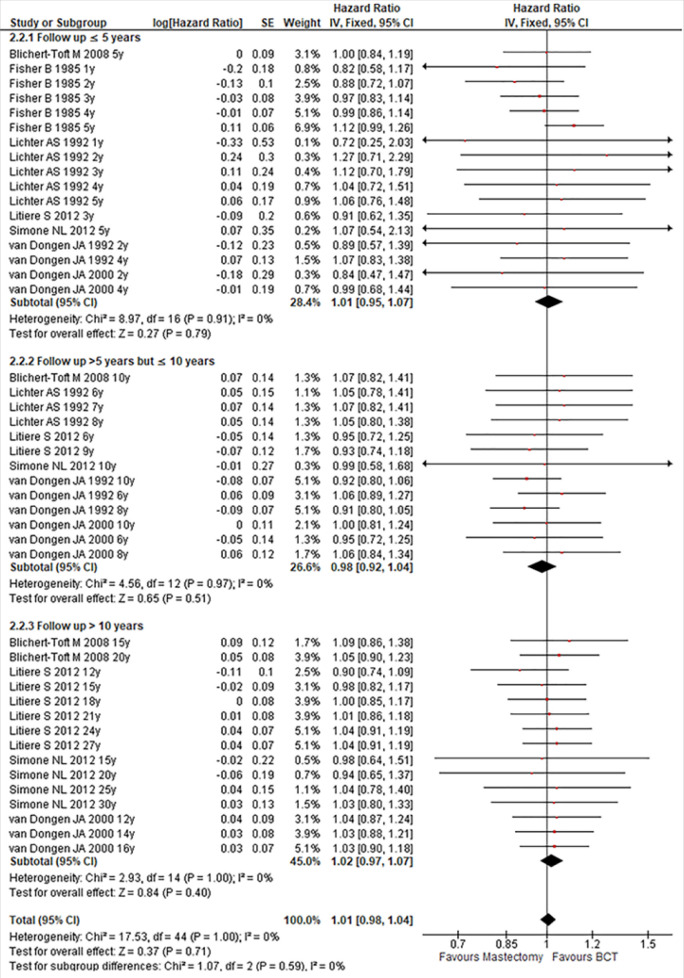
Forest Plot Comparing Mastectomy with BCT in Follow Up Period Subgrouping

In subgroups analysis, there was no any significant difference according to the follow up period. Their HRs and CI wee (HR:1.01; 95% CI:0.951-1.07; P:0.79), (HR:0.98; 95% CI:0.92-1.04; P:0.51) and (HR:1.02; 95% CI:0.97-1.07; P:0.40) respectively for up to 5 years or less, between 5 and 10 years and more than 10 years of follow up. In the three cases, there was no evidence of significant heterogeneity across studies. Their I^2^ and P were (I^2^:0%, P:0.91); (I^2^:0%, P:0.97); (I^2^:0%, P:1.00) respectively for up to 5 years or less, between 5 and 10 years and more than 10 years follow up as shown in figure 5.

### Risk of Bias

The most included studies had low risk of bias as assessed in figure 6 byusing the revised Cochrane risk-of-bias tool for randomised trials (RoB 2). Indeed, the red colour shows a high risk of bias and the yellow colour an intermediate risk when the green colour shows a low risk of bias, which is the case in this study.

## DISCUSSION

This meta-analysis summarised the OS of breast cancer patients at early stage when they are treated by mastectomy on one hand and when they are treated bb BCT on another hand. Moreover, it assessed the influence of follow up period on OS. This meta-analysis used two methods, one very commonly used (dichotomous) and another not popular (generic inverse variance). Interestingly, both arrived at the same conclusions.

In fact, it found that using either dichotomous method or generic inverse variance, there was no any significant difference between the two types of surgery in term of OS in general and in subgroup analysis especially. However, a recent meta-analysis concluded that mastectomy was benefit compared with BCT.^7^ We could thing that these disagreements are due to different methods used. In this case, this study has an advantage of having used two different methods which gave the same conclusions.

Cai X with his coleagues found that BCT was the better choice than MT for Chinese women with early-stage breast cancer even though they worked on non rendomized trials.^8^ The similar results were found by Vila J and colleagues. For them, mastectomy provides better OS compared to breast conserving surgery followed by whole breast radiotherapy in early breast cancer patients aged 40 years or younger.^6^ Note that they worked also on non randomised trials. At the contrary, other large population-based studies comparing breast-conserving surgery followed by radiation therapy with mastectomy supported that BCT might be good treatment in most breast cancer patients with early stage when both treatments are available.^30,31^

Considering what said above, this study contributed to clarify this point when randomised trials are involved even though the contribution is not enough for generalization. Since there are many cancer registries world wide, several studies comparing the OS between mastectomy and BCT should be found. Nevertheless, performing a metanalysis with many non randomised studies could provide another point of view.

This study used the data generated using the tool proposed by Tierney JF with his colleagues which facilitated to incorporate time-to-event data into meta-analysis.^10^ This tool was usefull because it allowed to know the log (HR) and its SEs at each level of assessment. This was not possible when used the dichotmous data. It could be evaluated in a large randomised trial to set up as software or to integrate it in the existing statistical softwares for meta-analysis.

## CONCLUSION

Even thought this study had many strengths such as the use of randomised trials, combination of two different methods, it had some limitations. We may mention a small number of included studies, variabilities in different trials' protocols which could affect somehow the outcome. Therefore, further studies are still needed to strengthen this findings. Meanwhile, this study shows that there was no significant difference between patients with early stage breast cancer when they are treated by mastectomy or BCT in term of overall survivals. Additionnally, the follow up period had no any influence on the both types of treatment in term of overall survivals. We suggest that BCT or mastectomy should be discussed between the care team and the patient, taking into account the financial means available to the patient especially in low-income countries, the benefits of the surgery and the patient's preferences.
